# A framework for the identification of long-term social avoidance in longitudinal datasets

**DOI:** 10.1098/rsos.170641

**Published:** 2017-08-02

**Authors:** Kasha Strickland, Alexis Levengood, Vivienne Foroughirad, Janet Mann, Ewa Krzyszczyk, Celine H. Frère

**Affiliations:** 1 Genecology Research Centre, University of the Sunshine Coast, Sippy Downs, Maroochydore DC, Queensland 4558, Australia; 2 Division of Marine Science and Conservation, Duke University Marine Laboratory, Beaufort, NC 28516, USA; 3 Department of Biology and Psychology, Georgetown University, Washington, DC 20057, USA

**Keywords:** avoidance, social behaviour, longitudinal data, spatial distribution, social preference

## Abstract

Animal sociality is of significant interest to evolutionary and behavioural ecologists, with efforts focused on the patterns, causes and fitness outcomes of social preference. However, individual social patterns are the consequence of both attraction to (preference for) and avoidance of conspecifics. Despite this, social avoidance has received far less attention than social preference. Here, we detail the necessary steps to generate a spatially explicit, iterative null model which can be used to identify non-random social avoidance in longitudinal studies of social animals. We specifically identify and detail parameters which will influence the validity of the model. To test the usability of this model, we applied it to two longitudinal studies of social animals (Eastern water dragons (*Intellegama lesueurii*) and bottlenose dolphins (*Tursiops aduncus*)) to identify the presence of social avoidances. Using this model allowed us to identify the presence of social avoidances in both species. We hope that the framework presented here inspires interest in addressing this critical gap in our understanding of animal sociality, in turn allowing for a more holistic understanding of social interactions, relationships and structure.

## 1. Introduction

Understanding social behaviour is a key component of behavioural and evolutionary ecology. Accordingly, the mechanisms driving social attraction and the adaptive significance of the subsequent social interactions have been well studied across numerous taxa (for examples, see [1–4]). As a result, we have gained extensive insight into how and why individuals associate with each other. However, at the core of sociality lies not only attraction to, hereafter social preference, but also avoidance of conspecifics [5–8]. Nevertheless, studies have primarily focused on social attraction, with a wide variety of metrics devoted to measuring association and affiliation [5,9,10], while social avoidance has received far less attention. Here, we define social avoidance by the relative absence of association between individuals that overlap spatially. This differs from a lack of social preference where individuals do not seek each other out, but may still show mutual tolerance and occasionally associate as a by-product of other relationships or spatial overlap. In social avoidance, we would predict that individuals would be likely either to leave or not join groups containing individuals that they avoid. Social avoidance may be unilateral or mutual. Avoidance of conspecifics is clearly as important as affiliation in that, like predator avoidance, conspecific avoidance necessarily incurs a heretofore unrecognized cost, one critical to the study of sociality and its evolution.

There are multiple ways in which individuals may choose to avoid conspecifics (see box 1), but social avoidance refers specifically to instances in which individuals share space but do not associate. To date, such avoidance behaviour has most frequently been studied in the context of inbreeding avoidance [11], sex segregation [12], avoidance of diseased conspecifics [13,14] and dominance hierarchies [15]. Avoidance has previously been measured largely as instances of an individual's immediate withdrawal from social contact with conspecifics [16–19]. For instance, a subordinate's immediate avoidance of and withdrawal from contact with approaching or aggressive individuals has been used to determine relative social status in dominance hierarchies [20]. However, as with affiliative social interactions, short-term avoidances may develop into long-term avoidances, where individuals consistently avoid certain conspecifics over time. For instance, we know that, under certain circumstances, short-term affiliative social interactions (e.g. grooming) can, after repeated interactions, develop into stable, social relationships [21–23]. Short-term avoidances such as those described above may, similarly, manifest into long-term avoidances, where individuals, given the spatio-temporal potential for repeated interactions, avoid one another consistently over time. Nevertheless, how, or indeed if, social avoidances are sustained over longer time periods remains unknown.

**Box 1.** Types of avoidance behaviour.

type of avoidance	definition
spatial avoidance	individuals avoid sharing any space with each other
short-term social avoidance	individuals immediately withdraw from interacting (either socially or aggressively) with another individual. The individuals may interact at a later stage
long-term social avoidance (spatio-temporal avoidance)	individuals avoid each other consistently over a long period of time. Despite sharing a significant portion of space, they either do not associate, or associate very little

Avoidance of conspecifics may occur in many social situations, and for a multitude of reasons. For example, Eastern chipmunks (*Tamias striatus*) with overlapping neighbouring territories avoid each other by adjusting their space use temporally, allowing the chipmunks to use the shared space at different times [24]. Current theory suggests that social animals simultaneously choose to associate with some conspecifics, while avoiding others within their social environment [6–8]. Such avoidances would therefore be expected to occur within the same spatial domain as social associations. In fact, a few studies of social animals have demonstrated that some individuals share much of their space, but do not associate, suggesting that individuals may be avoiding each other (e.g. [25,26]). However, in these studies, the social avoidances were largely identified as a by-product of investigating social preference. Moreover, while many methods that measure pairwise preference also tacitly measure avoidance, the validation of these methods is often with isolating preferences in mind. Consequently, while avoidance of conspecifics has been proposed as an important mechanism in the structuring of social associations [27], the quantitative investigation and identification of non-random social avoidances remains undefined and untested.

Both the presence and absence of social association can occur by chance. For example, animals that share a social environment, and the resources within it, may both encounter each other—or not—solely by chance due to overlapping patterns of space use [28]. Disentangling random processes from behavioural choice can be, and often is, done by comparing observed patterns of behaviour with null models [28–31]. Other authors have offered ways to measure social avoidances (e.g. [29,30]), such as by randomizing the membership of groups, or by using pairwise spatial overlap measures as a predictor of associations. However, we propose that the use of a spatially explicit null model is most appropriate. This is because, by accounting for individuals' observed space use and spatial heterogeneity, this method allows for more robust predictions of random association patterns [28]. In contrast to other methods which also use space, by randomizing individuals’ spatial positions, we are able to better predict patterns of association resulting from space use, thereby isolating instances where individuals would be expected to associate but do not (i.e. avoidance). To identify long-term social avoidances, we provide a general framework using a spatially explicit null association model and demonstrate its application to two species (Indo-Pacific bottlenose dolphins (*Tursiops aduncus*) and Eastern water dragons (*Intellegama lesueurii*)) with disparate ecology and social structure.

## 2. General framework

**Figure 1.**
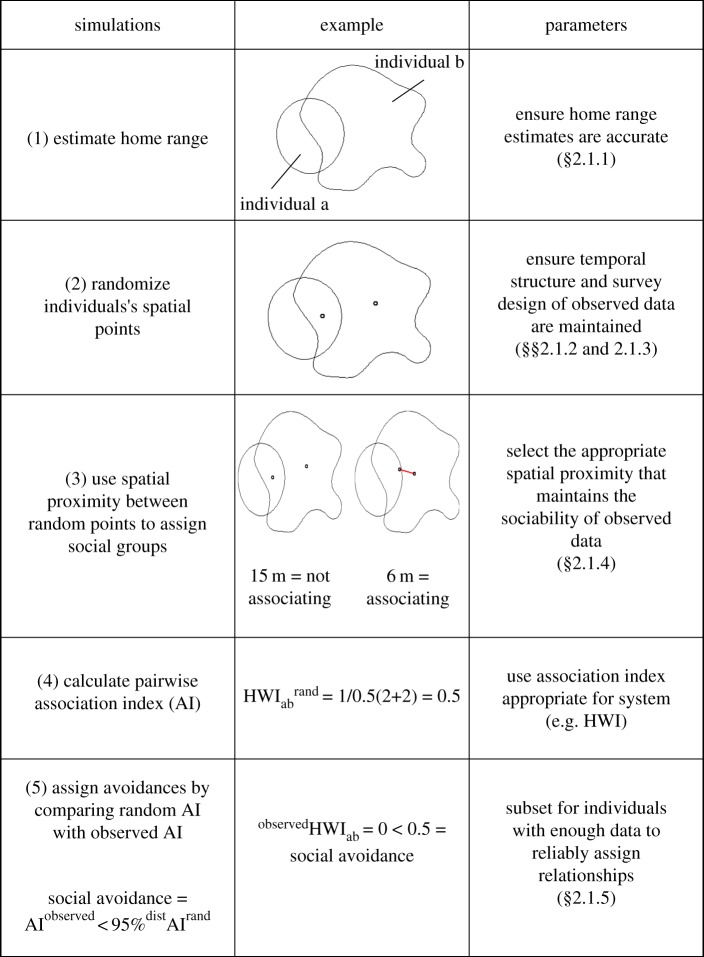
Diagram describing the steps involved with the simulations. The example, based on two individuals, illustrates the steps and the parameters that are important to consider. HWI is calculated as *N*_ab_/(*N*_ab_ + 0.5(*N*_a_ + *N*_b_) + *Y*_ab_) where *N*_ab_ is the number of times individuals *a* and *b* were seen associated, *N*_a_ and *N*_b_ are the number of times each individual was seen, respectively, and *Y*_ab_ is the number of times they were seen in the same sampling period but not associated.

The null association model used here produces expected social associations between dyads which result from expected use of space (figure 1). The model does so by randomizing individuals' spatial positions within their home ranges (the area in which individuals live), using either home range contours or utilization distributions (UDs). Random points are then generated according to individuals' UD (electronic supplementary material, figure SA1), as well as the probability of being sighted, and random pairwise associations are extracted based on proximity between random spatial positions (see figure 1 and §2.1.4 for how to identify this threshold). Spatial proximity between animals is a well-accepted method of estimating pairwise associations in studies of social species where interactions are infrequent and/or difficult to observe [32,33]. With both the observed and the randomly generated social groupings, the strength of the pairwise associations can then be estimated and compared using an appropriate association index for the study species (e.g. simple ratio index, twice-weight index, social affinity index). Here, we used the half-weight index (HWI) because it is the most suitable index for both systems (see [34]). The HWI is calculated using the following formula:

$$\text{HWI}=\frac{N_{\mathrm{ab}}}{N_{\mathrm{ab}}+0.5(N_{a}+N_{b})+Y_{\mathrm{ab}}},$$

where *N*_ab_ is the number of times individuals *a* and *b* were seen associated, and *N*_a_ and *N*_b_ are the number of times each individual was seen and *Y*_ab_ is the number of times they were seen in the same sampling period but not associated [34]. Applying this model iteratively allows us to generate a random probability distribution against which the observed value can be statistically compared. In other words, a *p* value for each dyad is calculated as the proportion of times the random association index was smaller (or greater for preferences) than the observed. Avoidances can therefore be defined as instances where 95% (or more) of random HWIs were greater than the observed HWI.

The null association model has been previously described elsewhere [25,35], and integrated in the *Digiroo2* package in R [36,37]. Here, we have identified critical parameters essential in the generation of a valid null model for social avoidance. Therefore, in this paper, we expand on the approach described by Carter *et al.* [25] in order to increase its applicability to other populations, and for its utility in detecting social avoidances.

### 2.1. Optimizing the null model

A framework is provided below with which to generate the null model, with attention to parameters that vary according to the species’ behavioural ecology, and the observation protocol used during data collection. These are important, as failing to incorporate them may lead to type I and/or type II errors.

#### 2.1.1. Utilization distribution

This spatial model randomizes individuals' locations according to their home range. Here, we suggest the use of individuals' UDs because this retains more information about individuals' variable use of space. More specifically, it accounts for the fact that individuals do not use their space evenly. UDs are home ranges that describe the frequency distribution of an individual's location data [38], estimating an individual's probability of occurrence within an area across a grid of the study sites’ coordinates. Accurate (i.e. stable) UD estimations depend on sample size, and identifying the minimum number of sightings necessary can be species- or population-specific. The data should include only individuals that meet the required minimum sightings.

#### 2.1.2. Temporal structure of data

When applying the model to longitudinal datasets, it is important for the null model to reflect the temporal structure of the data. This is because demographic events within the study population such as birth, death, immigration and emigration will affect the extent to which pairs of individuals overlap temporally in the observed data. This can be achieved, for instance, by restricting data in analyses to time periods in which there were few demographic changes (e.g. births/deaths). If restricting data is neither possible nor desirable, the model can be set up so that individuals are included in random sampling periods (e.g. 1 day) only if they overlap temporally. This may be especially important for difficult-to-observe species in which the observation period required to achieve accurate home range estimates extends over an amount of time in which demographic changes are unavoidable.

#### 2.1.3. Survey design

Null models must consider how the surveys (animal sighting records) were collected. During the simulations, individuals sighted within random sampling periods are randomly selected from the population; however, the survey design of some studies does not cover the entire study site in each sampling period. This would result in uneven sampling coverage which must be accounted for within the model.

#### 2.1.4. Reflecting population sociability

The null model must incorporate the amount of time individuals in the population spend together (e.g. sociability of the population), while randomizing the membership of groups or pairs formed. The sociability of populations can be estimated in several ways (e.g. average association index, average number of groups or average group size), and obtaining comparable levels of sociability in the null can also be done in several ways. Retaining group size and number of groups, as per the Manly–Bejder permutation method [39], may be applicable for group-living species (e.g. dolphins); however, a more general measure is needed when applying this method to species which do not live in clearly defined groups (e.g. water dragons). Therefore, due to the general applicability of the average association index (e.g. HWI), we suggest this measure as the most appropriate.

Animals do not move randomly. As such, when simulations move animals randomly within their home range (as in the null model), the number of social associations will be greatly reduced. As a result, to optimize the null model to retain the sociability of the population, we must manipulate certain parameters of the observed data. This can be done in a number of ways, but we focused on two major options: increasing the distance proxy used for social associations, or increasing the number of individuals sighted per random sampling period. However, increasing the sample size per sampling period would result in inflating the likelihood of dyads being sighted within the same sampling period. Because association indices such as the HWI are calculated based on pairwise sample size, any difference in dyads being sighted within the same sampling period would result in random pairwise HWIs which are no longer comparable to the observed HWI. Therefore, we suggest that to achieve comparable HWIs, the distance within which individuals are grouped together (*gprox*) should be adjusted. This will, in most instances, result in a larger *gprox* than is used in the observed data because, in most instances, using the same distance will result in underestimating the sociability of the population. This is also the most flexible approach, as it may be that a large proportion of the population were sighted within each sampling period, thus making increasing the number of individuals per random sampling period difficult.

#### 2.1.5. *Post hoc* selection of individuals

Estimates of social associations are influenced by the number of sightings each individual has. Moreover, the variance in sample size between individuals will affect the accuracy of the classification of that relationship [34]. Often this is accounted for, first by using appropriate association indices and, second, by only using individuals with sufficient sightings to accurately measure the population's sociability. When building the null model, we first used a subset of the data to include only individuals that had enough sightings to accurately estimate their UD (see §2.1.1). However, in most cases, to obtain reliable assignment of relationships (e.g. avoid—random—prefer), more sightings will be needed than that which is needed to gain accurate home range estimations. Therefore, for *post hoc* analysis we selected only individuals which had the minimum number of sightings required to classify pairwise relationships (e.g. avoidance—random/neutral—preference) accurately. We selected individuals for identification of avoidance (and preference) *post hoc* because, in the null model, it is important to retain as many individuals as possible in order to optimize the null accurately (i.e. to match observed sociability). Nonetheless, selecting individuals with enough social data post simulations results in confident identification of pairwise relationships.

### 2.2. Identifying avoidances and preferences

Avoidances are identified by randomizing groups according to individuals' UD, and then generating a distribution of random HWIs for each pair. Following this, a *p* value for each dyad is calculated as follows:

$$P=\sum_{j=1}^{B}\frac{Y_{j}\leq X}{B},$$

where *B* is the number of replicates, *Y* is the random HWI and *X* is the observed HWI [40]. Thus, the *p* value represents the proportion of times the random association index was smaller than the observed. Here, we classify pairwise avoidances as those that are seen together significantly less than would be expected if they moved randomly (with a 95% CI). Therefore, these avoidances can be instances of both HWI = 0, but also of HWI > 0. Preferences can also be identified by calculating a *p* value as the proportion of times the observed HWI is greater than in the null model (with a 95% CI).

The strength of pairwise avoidances may be assessed by using a measure of pairwise home range overlap. For example, a pair of individuals that avoid each other and share 100% of their home ranges could be seen to be a stronger social avoidance than in instances where a pair share 30% of their home ranges.

## 3. Case study: dragons and dolphins

We used longitudinal data from two social vertebrates (Eastern water dragons and bottlenose dolphins) to illustrate and test the framework's utility in identifying long-term social avoidances. The framework was applied using individuals' 100% UD, and then repeated using only data that fell within each animal's 50% UD to examine behaviour in the ‘core’ home range.

Eastern water dragons are a gregarious reptile, whose social dynamics resemble fission–fusion systems [41]. Males are plastic in their mating tactics (territoriality or satellite) [42] and, importantly, there are instances where pairs of individuals share large proportions of their home range but are never seen together [41]. To quantify social associations, a 1.85 m distance proxy is used [41]. This distance is used for three reasons. First, it was identified statistically by taking the upper first percentile of all pairwise geographical proximities [41]. Second, affiliative interactions require time within close proximity of one another [28,43–45]. Third, our observations have shown that individuals which do not tolerate each other will react aggressively to one another within this distance. Data are, however, filtered to remove those instances where aggressiveness is observed to ensure that close social proximity reflects social tolerance.

We used data collected as part of an ongoing behavioural study (2010–present) of a population of dragons at Roma St Parklands, Brisbane, Australia [46]. The population has an estimated size of 336 individuals (estimated using Jolly–Seber mark-recapture methods in SOCPROG v. 2.6 [30]). Behavioural surveys were conducted along the same transect of the Parklands, which covers approximately 85% of the population. Surveys were carried out twice a day (AM and PM), on average three times a week. This time period represents the time in which they are most active (i.e. not hibernating). For each individual sighted, we collected head profile photograph (using Canon EOS 600 digital camera), GPS coordinates (using GARMIN eTrex10 handheld device, accurate to 3 m), sex and observations of aggressive behaviour typical of agamid lizards (e.g. head bob, tail slap, arm wave, push-ups). Sex was assigned based on sexual dichromatism and sexual dimorphism present in the species [47]. Profile photographs were used to identify individuals using the I^3^S Manta software package [48] using unique scale patterns and coloration [46].

Bottlenose dolphins (*Tursiops aduncus*) live in open communities, exhibiting fission–fusion social organization, characterized by changing group membership through space and time [49–53]. In Shark Bay, Western Australia, males form stable long-term alliances [54–57], while females are more socially flexible, forming relatively loose, kin-based associations [26,49,58]. In Shark Bay, dolphins are bisexually philopatric and have large, overlapping temporally stable home ranges [59].

We used data from an ongoing, long-term (approx. 30 year) study of over 1600 bottlenose dolphins resident to the eastern gulf of Shark Bay, Western Australia. The main study site is approximately 300 km^2^ and is highly environmentally heterogeneous [60]. Data were collected using boat-based opportunistic surveys, where scan sampling [61,62] was used for the first five minutes to determine group composition. Individuals were identified using standard dorsal fin identification techniques [63]. Sex was determined either by the presence of a dependent calf, views of the genital area [49] and, where available, genetic sex determination [64]. Age was assigned by physical and behavioural characteristics [65,66]. Group membership was determined using a 10 m chain rule, where all individuals within 10 m of each other were considered to be associating [49]. Ecological data (e.g. GPS location, water depth, sea state and seafloor substrate) were recorded during each survey.

### 3.1. Optimizing the null model

We optimized the null model for both the full dataset, and a dataset restricted to the core home range (50% UD) in order to investigate differences in the presence of avoidance at different spatial scales.

#### 3.1.1. Utilization distribution

**Figure 2.**
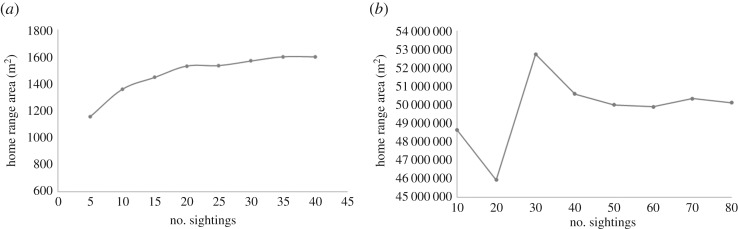
The effect of sample size on average home range size for dragons (*a*) and dolphins (*b*) illustrating that home range does not stabilize until sample sizes reach 25 and 45, respectively.

UDs were estimated using GPS relocations for each individual and an optimized smoothing parameter. Pairwise home range overlap was calculated using 95% and 50% volume of intersection (VI) [38]. VI is a commonly used index of pairwise home range overlap. It measures the amount of overlap between two UDs and calculates a value between 0 (no overlap) and 1 (complete overlap). All home range analyses were conducted using the *adehabitatHR* package within the R statistical environment [37,67]. To determine the minimum number of sightings required to gain accurate estimates of individuals' UD, we estimated home range size for the same individuals multiple times, randomly selecting sequentially increasing numbers of sightings (dragons from 5 to 40 sightings; dolphins from 20 to 70 sightings). We further measured pairwise VI with each set of randomly selected sightings and assessed correlations between these and VI estimated from the full data. In both dolphins and dragons, VI was very stable across sightings (see electronic supplementary material, table SA1). However, in order to be confident that we have measured home ranges accurately, we identified the minimum number of sightings required at which the size of the home range area stabilizes (i.e. stops increasing) (figure 2).

##### 3.1.1.1. Dragons

For dragons, a previously optimized smoothing parameter of seven metres [46] was used and a minimum number of 25 sightings was required for stable home range estimates (figure 2*a*). Although estimates at 20 and 25 sightings were comparable, we selected 25 sightings as a conservative estimate in order to increase the accuracy of home range measurements.

##### 3.1.1.2. Dolphins

For dolphins, UDs were estimated using an individual-specific reference bandwidth smoothing parameter [68], accounting for a land boundary (simplified from the coastline of the Peron peninsula) [69], as implemented in adehabitatHR [67]. Because our simplified boundary could not completely account for the tortuosity of the coastline, any remaining land area was removed from all final UDs and probability densities were re-standardized to 1. A minimum number of 45 sightings were required to gain stable home range estimates (figure 2*b*).

#### 3.1.2. Temporal structure of data

##### 3.1.2.1. Dragons

We used data collected between September 2015 and April 2016. This was because during this field season, we reliably captured enough sightings per individual. The data used in this study constituted 142 surveys (i.e. sampling periods), during which an average of 54 unique individuals (± 12 individuals) and 17 pairs (± 12 pairs) were sighted per survey.

##### 3.1.2.2. Dolphins

For this study, we used data collected during a period of 15 years (2001–2015). We used only survey data from May to September to reduce the influence of range shifts due to the breeding season [70,71]. All dependent calves, as well as any individual under 4 years old (average weaning age [72]) were omitted from analysis. If an individual was sighted more than once in a day, the last survey in which it was sighted was used to reduce spatial and temporal autocorrelation [59].

Because we used a 15-year dataset, we designed our model to account for demographic turnover caused by the entrance/exit of individuals within the population. This is important because failing to account for the temporal opportunity for pairs to interact can mask real social processes and structure [73]. Demographic turnover was accounted for in two ways. First, observed patterns of temporal overlap were incorporated within the null model by allowing only individuals who were available simultaneously to be included within the same random sampling period. Second, we only calculated HWIs over the period in which both animals were available to associate. We used conservative measures (i.e. last sighting date) to restrict animal associations in the absence of precise death dates.

#### 3.1.3. Survey design

##### 3.1.3.1. Dragons

Surveys conducted at Roma St Parklands cover the full transect during each sampling period. Therefore, we did not need to account for the spatial structure of the surveys within the null model.

##### 3.1.3.2. Dolphins

Owing to the size of the study site, the opportunistic surveying method and the necessity to begin all survey effort from the same launch point, our study site was unequally sampled. To account for this, we incorporated our spatial survey structure into our simulated data. This was done by creating daily minimum convex hulls with a 1 km buffer around each day's survey locations and the launch point (see electronic supplementary material, appendix figure SA2). Dolphin sightings were then randomly simulated within their UD according to the probability that each animal would be sighted within each daily area. The number of individual sightings in each sampling period was determined using a standard population density for the study site (0.45 dolphins km^−2^), and ranged from 2 to 40 animals per day (mean 9 ± 6).

#### 3.1.4. Reflecting population sociability

The null model for both dragons and dolphins was optimized by changing the pairwise geographical proximity threshold (*gprox*). We tested a series of distances in sequential increments equal to our grid cell size used in calculation of UD. It is important to ensure that the *gprox* value accurately groups individuals from appropriate social environments (e.g. within a biologically meaningful distance). Therefore, we ensured that the *gprox* distances that were tested were contained within the smallest observed centroid to contour distance. This ensures that the null's *gprox* value, though greater than the proximity used in the real data, is still biologically possible. The *gprox* value which produced a null model that best matched the observed average HWI was then selected for use in the final null model.

##### 3.1.4.1. Dragons

One hundred and eleven individuals (of 330) had at least 25 sightings and were therefore included in analyses. The null model randomized positions for 64 individuals in each of the 114 random sampling periods. This maintained the observed average number of individuals sighted per sampling period, and the number of sampling periods in the data. The optimal null model for dragons used a *gprox* value of five metres for the full home range, and four metres for the core home range (table 1).

**Table 1.** Optimizing the null model to match the average sociability of the population. This table presents the average half-weight association index (HWI) for the observed dataset, and from random iterations. Random iterations use increasing pairwise geographical proximity as a proxy for social associations (*gprox*). The *gprox* which matches the observed value was then used in the final iterations (shown in bold). HWI is calculated as *N*_ab_/(*N*_ab_ + 0.5(*N*_a_ + *N*_b_) + *Y*_ab_), where *N*_ab_ is the number of times individuals *a* and *b* were seen associated, *N*_a_ and *N*_b_ are the number of times each individual was seen, respectively, and *Y*_ab_ is the number of times they were seen in the same sampling period but not associated.

dragons			dolphins		
*gprox* (m)	full	core	*gprox* (m)	full	core
observed	**0.0034**	**0.0020**	observed	**0.0604**	**0.0525**
1	0.0001	0.0001	600	0.0145	0.0120
2	0.0004	0.0004	700	0.0214	0.0181
3	0.0010	0.0010	800	0.0307	0.0233
4	0.0018	**0.0018**	900	0.0416	0.0316
5	**0.0031**	0.0030	1000	**0.0543**	0.0397
6	0.0048	0.0047	1100	0.0672	**0.0488**
7	0.0071	0.0067	1200	0.0836	0.0576
8	0.0105	0.0090	1300	0.0956	0.0658
9	0.0147	0.0115	1400	0.1071	0.0717

##### 3.1.4.2. Dolphins

Seventy-six individuals (of 738) had at least 45 sightings and were therefore included in analyses. We simulated positions for individuals in each of the 809 sampling period days accounting for both temporal availability and survey effort. The optimal null model for the dolphins used a *gprox* value of 1000 m for the full home range and 1100 m for the core home range (table 1).

#### 3.1.5. *Post hoc* selection of individuals

To ensure that we accurately measured the sociality of pairs, we investigated how sighting frequency affected the stability of classifications (i.e. preference—random—avoidance). To do this, we repeated analyses with increased increments of randomly selected sightings per individual. Using these restricted datasets, we generated 100 simulations per increment and estimated pairwise *p* values for each pair. We then compared correlations of classifications generated from these different numbers of sightings with the full dataset to determine the minimum number of sightings needed to obtain reliable pairwise classifications (table 2).

**Table 2.** Correlations of association classification rates. Correlation (Spearman's) between the classification of associations (preference—random—avoidance) at different numbers of sightings with those generated from the full dataset. Sightings were randomly sampled from the same individuals. Full dataset included only individuals with at least 40 sightings (*n* = 73) for dragons, and 100 sightings (*n* = 25) for dolphins.

	number of sightings per individual	correlation (*r*^2^) to full dataset
dragons	10	0.859
	15	0.887
	20	0.903
	25	0.930
	30	0.943
	35	0.942
	40	0.964
dolphins	45	0.732
	50	0.760
	55	0.801
	60	0.839
	65	0.865
	70	0.895
	75	0.897
	80	0.908
	85	0.910
	90	0.928
	95	0.917
	100	0.927

##### 3.1.5.1. Dragons

Correlations of classifications generated with different numbers of sightings reached 0.9 at 20 sightings (table 2). However, the rate of type I errors (e.g. false avoidances) dropped at 30 sightings, indicating that at 30 sightings, we have reliably estimated pairwise sociality while also reducing any error rate (table 3).

**Table 3.** Error rates with increased number of sightings. Number of type I and type II errors, in comparison to the full dataset, for both preferences and avoidances when subsampling different numbers of sightings from the same individuals. Full dataset included only individuals with at least 40 sightings (*n* = 73) for dragons, and 100 sightings (*n* = 25) for dolphins.

		sightings																		
		dragons							dolphins											
error type		10	15	20	25	30	35	40	45	50	55	60	65	70	75	80	85	90	95	100
type I (false positive)	preferences	0	2	0	0	0	2	2	2	2	2	1	2	1	1	1	3	4	2	2
	avoidances	26	8	12	4	2	0	2	1	3	4	2	5	7	7	5	8	3	12	7
type II (false negative)	preferences	38	36	36	38	36	32	32	14	11	10	13	10	10	11	9	9	6	10	4
	avoidances	0	0	0	0	0	0	0	0	20	21	18	16	11	10	12	10	11	8	10

##### 3.1.5.2. Dolphins

Correlations of classification reached 0.89 at 70 sightings, and correlations did not increase significantly with more than 70 sightings (table 2). Interestingly, error rates were not sightings dependent for dolphins (table 3). This indicated that using 70 sightings produced reliable estimates of pairwise sociality.

Having optimized the null model for each species (table 1), we generated 1000 optimized random datasets for both the full dataset, and a dataset restricted to the core home range (50% UD). We then compared our random pairwise association values to those calculated from the observed data. Relationships were classified as ‘avoidance’ if they fell below the 95% distribution of random values, and ‘preference’ if they fell above it.

## 4. Results

### 4.1. Dragons

A total of 99 individuals had at least 30 sightings and were therefore included in the final analyses, resulting in 4703 dyads. We found four cases of pairwise avoidance when using data from the full dataset. Of these, three were male–female and the remaining were male–male pairs. When restricting analyses to the 50% core home range, we found 34 cases of pairwise avoidance. Of these, 47% (*n* = 16) were male–female, 23% (*n* = 7) were male–male and 30% (*n* = 11) were female–female pairs. In both cases, more preferences were found than avoidances (full = 199, core = 76). Similar to avoidances, the majority of preferences were male–female pairs at both the full and at the core home range.

### 4.2. Dolphins

A total of 48 individuals had at least 70 sightings and were included in the analyses, resulting in a total of 1073 pairs. We found 332 cases of pairwise avoidance in the full dolphin dataset. Of these, 61% (*n* = 202) were male–female, 13% (*n* = 45) were male–male and 26% (*n* = 85) were female–female dyads. When data were restricted to the core home range, we found 247 cases of pairwise avoidance. Similarly to the full range, the majority were male–female dyads (male–female: 63%, *n* = 156; male–male: 17%, *n* = 41; female–female: 20%, *n* = 50). In both cases, there were fewer preferences than avoidances: full = 216, core = 181.

**Figure 3.**
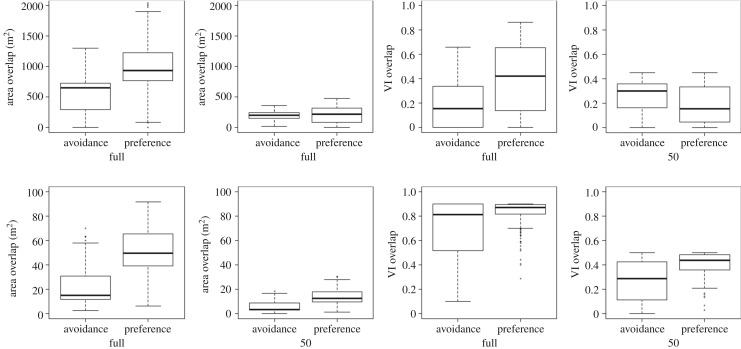
Boxplots showing the degree of pairwise overlap for dyads classified as both preference and avoidance in the full home range (full) and core home range (50), for dragons (top) and dolphins (bottom). Area overlap is the size of the intersection of each pair's home ranges. VI is volume of intersection home range overlap index.

For both dolphins and dragons, preferences and avoidances were found to occur within similar levels of shared space (figure 3). However, the levels of home range overlap that individuals that avoided each other shared were in the ranges of 10–90% (dolphins) and 5–50% (dragons) (figure 3). This indicates that some pairwise avoidances may be stronger than others.

## 5. Discussion

Despite being an integral part of an animal's social behaviour [6–8], long-term social avoidance has received little empirical attention. In this paper, we build on the framework initially presented by Carter *et al.* [25], addressing and implementing critical features of the data into the analysis of animals' social behaviour. The results, using data from two longitudinal studies, illustrate the utility of simulation-based methods, such as the method presented here, for isolating social avoidances.

The framework implemented in our model aims to predict associations assuming that animals move randomly within their home range. This has the advantage of accounting for the effect that space use has on pairwise associations [26,44]. Other methods for analysing social structure have also begun to suggest ways of accounting for the effect of space use. For example, Whitehead & James [29] suggested using pairwise spatial overlap measures (e.g. VI) as a predictor of associations when creating generalized affiliation indices (GAIs). Avoidances can then be isolated as instances where the GAI is in the lower fifth percentile (e.g. lower than expected given degree of spatial overlap). However, the advantage of our null model is that the spatial information used is of higher resolution, resulting in more robust predictions of random spatial associations and therefore providing greater precision in identifying pairwise avoidances. More specifically, when assigning avoidances based on the GAI, pairs of individuals with identical VI overlap and an HWI of zero would both be assigned as an avoidance. We, however, found that when identifying avoidances using the simulation model, dyads with similar VI and an HWI of, for example, zero were not all considered as avoidances. This is because the size of the shared area is taken into account, and, as such, pairs with the same VI and an HWI of zero may not both be assigned as avoidances.

In agreement with Best *et al.* [35], we found that the most reliable way to optimize the null model was to alter the grouping distance proxy used within the model to ensure the null model matched the observed level of sociability. In dolphins, this distance was slightly larger at the core range than at the full range. This may be because there are fewer social associations within dolphins' core ranges in comparison to their full range; thus, to obtain comparable levels of associations in the null model, the distance must be increased. Alternatively, it may be that there is less overlap between dolphins' core home ranges. This would also result in a greater distance proxy required to group individuals in the null model. In both species, however, altering this proxy resulted in a grouping distance larger than is used in the observed data. This indicates that, as expected, individuals do not move randomly. For example, the distribution of resources influences how individuals move within their home range. Similarly, an individual's movement may also be influenced by the distribution of conspecifics; in particular, individuals' spatial distribution may not only influence social interactions, but may also, in part, be determined by them. Disentangling whether the formation of social relationships is caused by overlapping spatial distributions, or overlapping distributions are caused by social relationships would be extremely challenging. Some might argue that social relationships should therefore be assessed without controlling for spatial activity (e.g. using the Manly–Bejder permutations implemented in the SOCPROG software [30,39]). However, in dolphins, for instance, social preferences have been shown to exist despite very little spatial overlap [59,60]. Moreover, the confounded effects of spatial distribution and social relationships would not affect the identification of social avoidances, given that these are identified as pairs of individuals which do not associate despite sharing significant portions of their home range.

In addition to considerations suggested by Best *et al*. [35], we identified other key parameters, not previously detailed by these authors, paramount to the correct use of the model. These included, for instance, maintaining the sample size within each sampling period, which is an integral feature of the Manly–Bejder [39] well-established permutation technique. Another key parameter identified was accounting for temporal overlap of individuals, which has also been discussed conceptually elsewhere [29], but not yet fully implemented (but see [52]). Accounting for each of the parameters detailed within this framework is important because slight violations will result in spurious results. Therefore, the strength of the framework we present here lies in both the unification, and extension of, principles independently used within established methodologies.

Here, we show that, as theory suggests (e.g. [5,7]), individuals of both species avoid and preferentially associate within the same space. This indicates that, similar to Eastern chipmunks [24], non-random social avoidances occur in both space and time (i.e. they share space but not at the same time). Interestingly, we found that avoidances in dragons primarily existed within their core home range. Dragons are territorial animals [42,47], and so it may be that the core home range may better represent the individuals’ territories, and therefore their primary social environment. Further investigation is needed to explore such ideas. In contrast, bottlenose dolphins live in an open social network (where affiliative interactions extend beyond the individuals’ social group to members of other groups) [70] with high levels of home range overlap, suggesting that avoidances will occur throughout individuals’ full home ranges.

We present the first evidence, to our knowledge, for the presence of long-term social avoidances measured across multiple species using the same framework. The causality of the social avoidances identified here is probably diverse. For instance, it may be that some avoidances are caused by the need to avoid conflict (e.g. avoidance of large or aggressive individuals). Alternatively, some avoidances may be driven by groups of individuals (e.g. ages of sexes) using space and/or resources at different times. Even so, the study of avoidance has hitherto been unexplored, and in using this framework to identify social avoidances, future research can focus on exploring the biology, and causality, of the avoidances found here. In addition, studying avoidance behaviour means to approach the study of social behaviour holistically, inclusive of both components of an individual's sociality (e.g. attraction/avoidance). A few methodologies (e.g. [30]) have suggested such an approach, yet this is rarely implemented in full, often without consideration of space use, which results in a somewhat skewed view of animal social behaviour. While at this stage we cannot elucidate either the directionality (i.e. who avoids whom) or the biological significance of the social avoidances we have identified, the framework provided can be used in addressing this critical gap in our understanding of animal social behaviour. We hope that this paper stimulates interest in a shift in the approach to studying social behaviour through inclusion of both avoidance and affiliative social associations.

## Supplementary Material

Figure of two dolphins utilisation distributions and randomised spatial positions; Figure of daily MCPs; Correlations of home range overlap generated with increasing numbers of sightings per individual

## Data accessibility

Data and R code are available from the Dryad Digital Repository: http://dx.doi.org/10.5061/dryad.tn36j [74]. Data and R code for the dolphin project can also be found at https://github.com/vjf2/digidolph.

## References

[1] Hamilton WD 1964 The genetical evolution of social behaviour. I. J. Theor. Biol. 7, 1–16. (doi:10.1016/0022-5193(64)90038-4) 5875341 10.1016/0022-5193(64)90038-4

[2] Schülke O, Bhagavatula J, Vigilant L, Ostner J 2010 Social bonds enhance reproductive success in male macaques. Curr. Biol. 20, 2207–2210. (doi:10.1016/j.cub.2010.10.058) 21093261 10.1016/j.cub.2010.10.058

[3] Hofmann HA et al. 2014 An evolutionary framework for studying mechanisms of social behavior. Trends Ecol. Evol. 29, 581–589. (doi:10.1016/j.tree.2014.07.008) 25154769 10.1016/j.tree.2014.07.008

[4] Clutton-Brock T 2009 Cooperation between non-kin in animal societies. Nature 462, 51–57. (doi:10.1038/nature08366) 19890322 10.1038/nature08366

[5] Lusseau D, Whitehead H, Gero S 2008 Incorporating uncertainty into the study of animal social networks. Anim. Behav. 75, 1809–1815. (doi:10.1016/j.anbehav.2007.10.029)

[6] Buijs S, Keeling LJ, Vangestel C, Baert J, Vangeyte J, Tuyttens FAM 2011 Assessing attraction or avoidance between rabbits: comparison of distance-based methods to analyse spatial distribution. Anim. Behav. 82, 1235–1243. (doi:10.1016/j.anbehav.2011.08.019)

[7] Ward A, Webster M 2016 Sociality: the behaviour of group-living animals, pp. 1–8. Cham, Switzerland: Springer International Publishing.

[8] Jolles JW, Fleetwood-Wilson A, Nakayama S, Stumpe MC, Johnstone RA, Manica A 2015 The role of social attraction and its link with boldness in the collective movements of three-spined sticklebacks. Anim. Behav. 99, 147–153. (doi:10.1016/j.anbehav.2014.11.004) 25598543 10.1016/j.anbehav.2014.11.004 PMC4289919

[9] Farine DR et al. 2015 The role of social and ecological processes in structuring animal populations: a case study from automated tracking of wild birds. R. Soc. open sci. 2, 150057 (doi:10.1098/rsos.150057) 26064644 10.1098/rsos.150057 PMC4448873

[10] Whitehead H 2008 Analyzing animal societies: quantitative methods for vertebrate social analysis. Chicago, IL: University of Chicago Press.

[11] Archie EA, Hollister-Smith JA, Poole JH, Lee PC, Moss CJ, Maldonado JE, Fleischer RC, Alberts SC 2007 Behavioural inbreeding avoidance in wild African elephants. Mol. Ecol. 16, 4138–4148. (10.1111/j.1365-294X.2007.03483.x) 17784925 10.1111/j.1365-294X.2007.03483.x

[12] Wielgus RB, Bunnell FL 1994 Sexual segregation and female grizzly bear avoidance of males. J. Wildl. Manage. 58, 405–413. (doi:10.2307/3809310)

[13] Behringer DC, Butler MJ, Shields JD 2006 Ecology: avoidance of disease by social lobsters. Nature 441, 421 (doi:10.1038/441421a) 16724051 10.1038/441421a

[14] Loehle C 1995 Social barriers to pathogen transmission in wild animal populations. Ecology 76, 326–335. (doi:10.2307/1941192)

[15] Carter AJ, Tico MT, Cowlishaw G 2016 Sequential phenotypic constraints on social information use in wild baboons. Elife 5, e13125 (doi:10.7554/eLife.13125) 27067236 10.7554/eLife.13125 PMC4829417

[16] Kutsukake N, Clutton-Brock TH 2008 Do meerkats engage in conflict management following aggression? Reconciliation, submission and avoidance. Anim. Behav. 75, 1441–1453. (doi:10.1016/j.anbehav.2007.09.018)

[17] Sommer V, Denham A, Little K 2002 Postconflict behaviour of wild Indian langur monkeys: avoidance of opponents but rarely affinity. Anim. Behav. 63, 637–648. (doi:10.1006/anbe.2001.1897)

[18] Iwasaki M, Delago A, Nishino H, Aonuma H 2006 Effects of previous experience on the agonistic behaviour of male crickets, *Gryllus bimaculatus*. Zoolog. Sci. 23, 863–872. (doi:10.2108/zsj.23.863) 17116989 10.2108/zsj.23.863

[19] Sueur C, Petit O 2008 Organization of group members at departure is driven by social structure in Macaca. Int. J. Primatol. 29, 1085–1098. (doi:10.1007/s10764-008-9262-9)

[20] Dewsbury DA 1982 Dominance rank, copulatory behavior, and differential reproduction. Q. Rev. Biol. 57, 135–159. (doi:10.1086/412672) 7051088 10.1086/412672

[21] Silk JB, Beehner JC, Bergman TJ, Crockford C, Engh AL, Moscovice LR, Wittig RM, Seyfarth RM, Cheney DL 2010 Strong and consistent social bonds enhance the longevity of female baboons. Curr. Biol. 20, 1359–1361. (doi:10.1016/j.cub.2010.05.067) 20598541 10.1016/j.cub.2010.05.067

[22] Silk JB, Beehner JC, Bergman TJ, Crockford C, Engh AL, Moscovice LR, Wittig RM, Seyfarth RM, Cheney DL 2010 Female chacma baboons form strong, equitable, and enduring social bonds. Behav. Ecol. Sociobiol. 64, 1733–1747. (doi:10.1007/s00265-010-0986-0) 20976293 10.1007/s00265-010-0986-0 PMC2952770

[23] Gomes CM, Mundry R, Boesch C 2009 Long-term reciprocation of grooming in wild West African chimpanzees. Proc. R. Soc. B 276, 699–706. (doi:10.1098/rspb.2008.1324) 10.1098/rspb.2008.1324 PMC2660945 18957365

[24] Getty T 1981 Territorial behavior of eastern chipmunks (*Tamias striatus*): encounter avoidance and spatial time-sharing. Ecology 62, 915–921. (doi:10.2307/1936989)

[25] Carter AJ, Macdonald SL, Thomson VA, Goldizen AW 2009 Structured association patterns and their energetic benefits in female eastern grey kangaroos, *Macropus giganteus*. Anim. Behav. 77, 839–846. (doi:10.1016/j.anbehav.2008.12.007)

[26] Frère C, Krützen M, Mann J, Watson-Capps J, Tsai Y, Patterson E, Connor R, Bejder L, Sherwin W 2010 Home range overlap, matrilineal and biparental kinship drive female associations in bottlenose dolphins. Anim. Behav. 80, 481–486. (doi:10.1016/j.anbehav.2010.06.007)

[27] Aplin L, Farine D, Morand-Ferron J, Cole E, Cockburn A, Sheldon B 2013 Individual personalities predict social behaviour in wild networks of great tits (*Parus major*). Ecol. Lett. 16, 1365–1372. (doi:10.1111/ele.12181) 24047530 10.1111/ele.12181

[28] Spiegel O, Leu ST, Sih A, Bull CM 2016 Socially interacting or indifferent neighbours? Randomization of movement paths to tease apart social preference and spatial constraints. Methods Ecol. Evol. 7, 971–979. (doi:10.1111/2041-210X.12553)

[29] Whitehead H, James R 2015 Generalized affiliation indices extract affiliations from social network data. Methods Ecol. Evol. 6, 836–844. (doi:10.1111/2041-210X.12383)

[30] Whitehead H 2009 SOCPROG programs: analysing animal social structures. Behav. Ecol. Sociobiol. 63, 765–778. (doi:10.1007/s00265-008-0697-y)

[31] Whitehead HAL 1997 Analysing animal social structure. Anim. Behav. 53, 1053–1067. (doi:10.1006/anbe.1996.0358)

[32] Whitehead H, Dufault S 1999 Techniques for analyzing vertebrate social structure using identified individuals: review. Adv. Study Behav. 28, 33 (doi:10.1016/S0065-3454(08)60215-6)

[33] Franks DW, Ruxton GD, James R 2010 Sampling animal association networks with the gambit of the group. Behav. Ecol. Sociobiol. 64, 493–503. (doi:10.1007/s00265-009-0865-8)

[34] Cairns SJ, Schwager SJ 1987 A comparison of association indices. Anim. Behav. 35, 1454–1469. (doi:10.1016/S0003-3472(87)80018-0)

[35] Best EC, Dwyer RG, Seddon JM, Goldizen AW 2014 Associations are more strongly correlated with space use than kinship in female eastern grey kangaroos. Anim. Behav. 89, 1–10. (doi:10.1016/j.anbehav.2013.12.011)

[36] Dwyer R, Best E, Goldizen A 2013 Digiroo2: an application programming interface for generating null models of social contact based on individuals’ space use. R package version 0.5.

[37] R Development Core Team. 2010 R: a language and environment for statistical computing. Vienna, Austria: R Foundation for Statistical Computing.

[38] Fieberg J, Kochanny CO 2005 Quantifying home-range overlap: the importance of the utilization distribution. J. Wildl. Manage. 69, 1346–1359. (doi:10.2193/0022-541X(2005)69[1346:QHOTIO]2.0.CO;2)

[39] Bejder L, Fletcher D, Bräger S 1998 A method for testing association patterns of social animals. Anim. Behav. 56, 719–725. (doi:10.1006/anbe.1998.0802) 9784222 10.1006/anbe.1998.0802

[40] Ruxton GD, Neuhäuser M 2013 Improving the reporting of P-values generated by randomization methods. Methods Ecol. Evol. 4, 1033–1036. (doi:10.1111/2041-210X.12102)

[41] Strickland K, Gardiner R, Schultz AJ, Frère CH 2014 The social life of eastern water dragons: sex differences, spatial overlap and genetic relatedness. Anim. Behav. 97, 53–61. (doi:10.1016/j.anbehav.2014.08.009)

[42] Baird TA, Baird TD, Shine R 2012 Aggressive transition between alternative male social tactics in a long-lived Australian dragon (*Physignathus lesueurii*) living at high density. PLoS ONE 7, e41819 (doi:10.1371/journal.pone.0041819) 22905109 10.1371/journal.pone.0041819 PMC3414507

[43] Hubalek Z 1982 Coefficients of association and similarity, based on binary (presence-absence) data: an evaluation. Biol. Rev. 57, 669–689. (doi:10.1111/j.1469-185X.1982.tb00376.x)

[44] Carter KD, Seddon JM, Frère CH, Carter JK, Goldizen AW 2013 Fission–fusion dynamics in wild giraffes may be driven by kinship, spatial overlap and individual social preferences. Anim. Behav. 85, 385–394. (doi:10.1016/j.anbehav.2012.11.011)

[45] Zhang P, Li B-G, Qi X-G, MacIntosh AJJ, Watanabe K 2012 A proximity-based social network of a group of Sichuan snub-nosed monkeys (*Rhinopithecus roxellana*). Int. J. Primatol. 33, 1081–1095. (doi:10.1007/s10764-012-9608-1)

[46] Gardiner RZ, Doran E, Strickland K, Carpenter-Bundhoo L, Frère C 2014 A face in the crowd: a non-invasive and cost effective photo-identification methodology to understand the fine scale movement of eastern water dragons. PLoS ONE 9, e96992 (doi:10.1371/journal.pone.0096992) 24835073 10.1371/journal.pone.0096992 PMC4024003

[47] Thompson M 1993 Estimate of the population structure of the eastern water dragon, *Physignathus lesueurii* (Reptilia: Agamidae), along riverside habitat. Wildl. Res. 20, 613–619. (doi:10.1071/WR9930613)

[48] Van Tienhoven AM, Den Hartog JE, Reijns RA, Peddemors VM 2007 A computer-aided program for pattern-matching of natural marks on the spotted raggedtooth shark *Carcharias taurus*. J. Appl. Ecol. 44, 273–280. (doi:10.1111/j.1365-2664.2006.01273.x)

[49] Smolker RA, Richards AF, Connor RC, Pepper JW 1992 Sex differences in patterns of association among Indian Ocean bottlenose dolphins. Behaviour 123, 38–69. (doi:10.1163/156853992X00101)

[50] Connor RC, Wells R, Mann J, Read A 2000 The bottlenose dolphin: social relationships in a fission-fusion society. In Cetacean societies: field studies of whales and dolphins (eds Mann J, Connor R, Tyack P, Whitehead H), p. 448 London, UK: University of Chicago Press.

[51] Aureli F et al. 2008 Fission-fusion dynamics: new research frameworks. Curr. Anthropol. 49, 627–654. (doi:10.1086/586708)

[52] Mann J, Stanton MA, Patterson EM, Bienenstock EJ, Singh LO 2012 Social networks reveal cultural behaviour in tool-using using dolphins. Nat. Commun. 3, 980 (doi:10.1038/ncomms1983) 22864573 10.1038/ncomms1983

[53] Wells RS, Scott MD, Irvine AB 1987 The social structure of free-ranging bottlenose dolphins. In Current mammalogy (ed. Genoways HH), pp. 247–305. Boston, MA: Springer.

[54] Connor RC, Smolker RA, Richards AF 1992 Two levels of alliance formation among male bottlenose dolphins (*Tursiops* sp.). Proc. Natl Acad. Sci. USA 89, 987–990. (doi:10.1073/pnas.89.3.987) 11607275 10.1073/pnas.89.3.987 PMC48370

[55] Connor RC, Smolker RA, Richards AF 1992 Dolphin alliances and coalitions. In Coalitions and alliances in humans and other animals (eds Harcourt AH, de Waal FBM), pp. 415–443. Oxford, UK: Oxford University Press.

[56] Connor RC 2010 Cooperation beyond the dyad: on simple models and a complex society. Phil. Trans. R. Soc. B 365, 2687–2697. (doi:10.1098/rstb.2010.0150) 20679112 10.1098/rstb.2010.0150 PMC2936175

[57] Connor RC, Heithaus MR, Barre LM 1999 Superalliance of bottlenose dolphins. Nature 397, 571–572. (doi:10.1038/17501)

[58] Gibson QA, Mann J 2008 The size, composition and function of wild bottlenose dolphin (*Tursiops* sp.) mother-calf groups in Shark Bay, Australia. Anim. Behav. 76, 389–405. (doi:10.1016/j.anbehav.2008.01.022)

[59] Tsai YJJ, Mann J 2013 Dispersal, philopatry, and the role of fission-fusion dynamics in bottlenose dolphins. Mar. Mamm. Sci. 29, 261–279. (10.1111/j.1748-7692.2011.00559.x)

[60] Sargeant BL, Wirsing AJ, Heithaus MR, Mann J 2007 Can environmental heterogeneity explain individual foraging variation in wild bottlenose dolphins (*Tursiops* sp.)? Behav. Ecol. Sociobiol. 61, 679–688. (doi:10.1007/s00265-006-0296-8)

[61] Altmann J 1974 Observational study of behavior—sampling methods. Behaviour 49, 227–267. (doi:10.1163/156853974X00534) 4597405 10.1163/156853974x00534

[62] Mann J 1999 Behavioral sampling methods for cetaceans: a review and critique. Mar. Mamm. Sci. 15, 102–122. (doi:10.1111/j.1748-7692.1999.tb00784.x)

[63] Würsig B, Würsig M 1977 Photographic determination of group-size, composition, and stability of coastal porpoises (*Tursiops truncatus*). Science 198, 755–756. (doi:10.1126/science.198.4318.755)

[64] Krützen M, Barre LM, Connor RC, Mann J, Sherwin WB 2004 ‘O father: where art thou?’—Paternity assessment in an open fission-fusion society of wild bottlenose dolphins (*Tursiops* sp.) in Shark Bay, Western Australia. Mol. Ecol. 13, 1975–1990. (doi:10.1111/j.1365-294X.2004.02192.x) 15189218 10.1111/j.1365-294X.2004.02192.x

[65] Mann J, Smuts B 1999 Behavioral development in wild bottlenose dolphin newborns (*Tursiops* sp.). Behaviour 136, 529–566. (doi:10.1163/156853999501469)

[66] Krzyszczyk E, Mann J 2012 Why become speckled? Ontogeny and function of speckling in Shark Bay bottlenose dolphins (*Tursiops* sp.). Mar. Mamm. Sci. 28, 295–307. (doi:10.1111/j.1748-7692.2011.00483.x)

[67] Calenge C 2006 The package ‘adehabitat’ for the R software: a tool for the analysis of space and habitat use by animals. Ecol. Modell. 197, 516–519. (doi:10.1016/j.ecolmodel.2006.03.017)

[68] Worton BJ 1995 Using Monte Carlo simulation to evaluate kernel-based home range estimators. J. Wildl. Manage. 63, 794–800. (doi:10.2307/3801959)

[69] Benhamou S, Cornélis D 2010 Incorporating movement behavior and barriers to improve kernel home range space use estimates. J. Wildl. Manage. 74, 1353–1360. (doi:10.1111/j.1937-2817.2010.tb01257.x)

[70] Randić S, Connor RC, Sherwin WB, Krützen M 2012 A novel mammalian social structure in Indo-Pacific bottlenose dolphins (*Tursiops* sp.): complex male alliances in an open social network. Proc. R. Soc. B 279, 20120264 (doi:10.1098/rspb.2012.0264) 10.1098/rspb.2012.0264 PMC3385473 22456886

[71] Wallen MM, Patterson EM, Krzyszczyk E, Mann J 2016 The ecological costs to females in a system with allied sexual coercion. Anim. Behav. 115, 227–236. (doi:10.1016/j.anbehav.2016.02.018)

[72] Mann J, Connor RC, Barre LM, Heithaus MR 2000 Female reproductive success in bottlenose dolphins (*Tursiops* sp.): life history, habitat, provisioning, and group-size effects. Behav. Ecol. 11, 210–219. (doi:10.1093/beheco/11.2.210)

[73] Cantor M, Wedekin LL, Guimarães PR, Daura-Jorge FG, Rossi-Santos MR, Simoes-Lopes PC 2012 Disentangling social networks from spatiotemporal dynamics: the temporal structure of a dolphin society. Anim. Behav. 84, 641–651. (doi:10.1016/j.anbehav.2012.06.019)

[74] Strickland K, Levengood A, Foroughirad V, Mann J, Krzyszczyk E, Frère CH 2017 Data from: A framework for the identification of long-term social avoidance in longitudinal datasets. Dryad Digital Repository. (http://dx.doi.org/10.5061/dryad.tn36j) 10.1098/rsos.170641 PMC5579122 28879006

