# Correction to: A framework for the identification of long-term social avoidance in longitudinal datasets

**DOI:** 10.1098/rsos.220017

**Published:** 2022-01-19

**Authors:** K. Strickland, A. Levengood, V. Foroughirad, J. Mann, E. Krzyszczyk, C. H. Frère

*R. Soc. Open Sci.* **4**, 170641 (Published Online 2 August 2017) (doi:10.1098/rsos.170641).

In this article, we tested the use of a framework for identifying social avoidances using data from two individual-based longitudinal studies. Upon publication of the article, we archived the two datasets used for analysis in the dryad repository (https://doi.org/10.5061/dryad.tn36j). When downloading these data recently, we realized that for one of the datasets that was uploaded to dryad (on eastern water dragons), some observations of individuals appear duplicated within surveys. We then returned to our records to investigate where this error occurred, and whether it had been present during analyses of our data and that are presented in the article. We discovered that the duplicated observations were not present in the data during analyses, and therefore do not affect the results presented in article. Instead, we found that the duplications were introduced in error during the formatting of data for archiving in dryad.

When preparing data for archiving, we aimed to upload the spatial data for the individuals and observations that were used, which is the data needed to replicate our results in the article. In the article, we use data that were subset from the larger longitudinal dataset to be from one breeding year (2015–2016) and for individuals that had at least 25 sightings. We used these spatial data to estimate pairwise associations, which are pairs of individuals within 1.85 m of each other. During the process of estimated pairwise associations, data is reformatted from a format where each row is one spatial observation of an individual in a survey, to a format which records each pairwise association, such that each row represents one association. As such, if an individual is within 1.85 m of more than one individual on a survey, it will have more than one association in that survey, and, in the data, that individual will therefore have more than one row on that given survey (to reflect each observation). This is the data that we used to estimate association indices (HWI) in our framework. As such, when we were preparing the data for dryad, we outputted data in this format, and then aimed to reformat it to reflect just the spatial locations per individual per day. However, during this process, we made an error and did not remove the observations that appear as ‘duplicated’ observations of individuals per survey, which would be the correct data that would be needed to reproduce our analyses. As such, the analyses presented in the article are unaffected, but the data that was archived would not be able to reproduce our results. The data are corrected now.

