# Supplementary material for: A framework for the identification of long-term social avoidance in longitudinal datasets
Source: R Soc Open Sci. 2017 Aug 2;4(8):170641. doi: 10.1098/rsos.170641 (PMC5579122; doi:10.1098/rsos.170641)
Supplement: Figure of two dolphins utilisation distributions and randomised spatial positions; Figure of daily MCPs; Correlations of home range overlap generated with increasing numbers of sightings per individual [file rsos170641supp1.docx]

Supplementary material: A framework for the identification of long-term social avoidance in longitudinal datasets


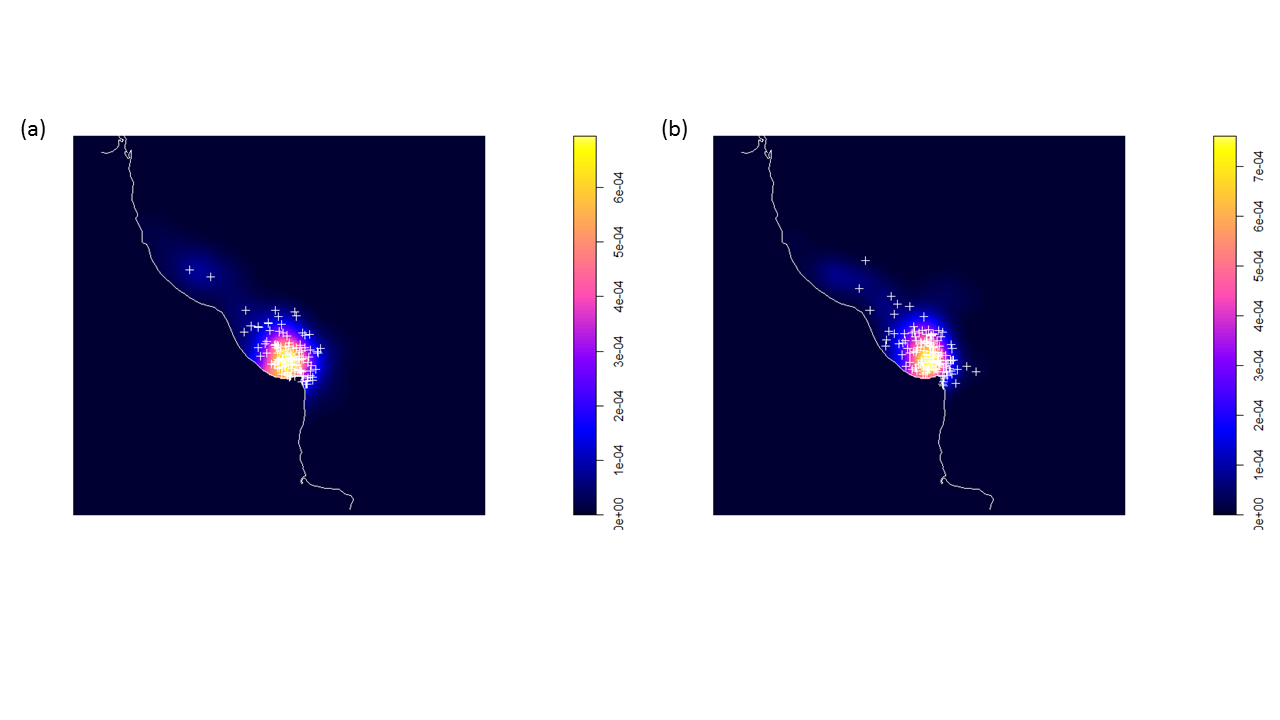


Fig A1. Randomised points created by *digiroo2,* overlaid on utilisation distributions, for two individual dolphins. Colour gradient represents the probability distribution of the individual being sighted at different coordinates.


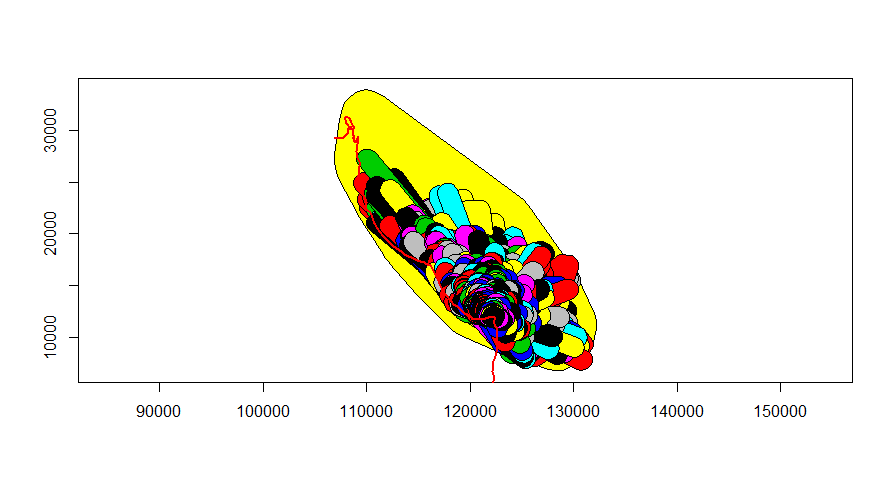


Figure A2. Figure showing minimum daily convex hulls used to standardise the null model to reflect sampling effort for a population of bottlenose dolphins in Shark Bay, Western Australia. Red line represents land boundary. Largest yellow polygon is entire study site. Smaller, overlapping polygons are daily convex hulls.

Table A1. Correlations of home range overlap generated with increasing numbers of sightings per individual

|  | Dragons | | |  |  |  |  |  |  | Dolphins | | |  |  |  |  |  |
| --- | --- | --- | --- | --- | --- | --- | --- | --- | --- | --- | --- | --- | --- | --- | --- | --- | --- |
| Sightings | 5 | 10 | 15 | 20 | 25 | 30 | 35 | 40 |  | 35 | 40 | 45 | 50 | 55 | 60 | 65 | 70 |
|  |  |  |  |  |  |  |  |  |  |  |  |  |  |  |  |  |  |
| Correlation to full | 0.95 | 0.97 | 0.98 | 0.98 | 0.99 | 0.99 | 0.99 | 0.99 |  | 0.94 | 0.95 | 0.96 | 0.96 | 0.97 | 0.98 | 0.98 | 0.98 |

Home range overlap calculated as volume of intersection (VI) for both dragons and dolphins. Sightings randomly selected for the same individuals at each increment, sequentially increasing the number of sightings randomly selected.
